# Comparison of gross pathology inspection and 9.4 T magnetic resonance imaging in the evaluation of radiofrequency ablation lesions in the left ventricle of the swine heart

**DOI:** 10.3389/fphys.2022.834328

**Published:** 2022-10-19

**Authors:** Eva Odehnalová, Lucia Valíková, Guido Caluori, Tomáš Kulík, Veronika Římalová, Tomasz Jadczyk, Eva Dražanová, Iveta Pavlova, Martin Pešl, Václav Kubeš, Zdeněk Stárek

**Affiliations:** ^1^ Interventional Cardiac Electrophysiology Group, International Clinical Research Center, St. Anne’s University Hospital Brno, Brno, Czech; ^2^ Nanotechnology, CEITEC Masaryk University, Brno, Czech; ^3^ IHU LIRYC, Electrophysiology and Heart Modeling Institute, Fondation Bordeaux Université, Pessac, France; ^4^ University Bordeaux, INSERM, Cardiothoracic Research Center of Bordeaux, Pessac, France; ^5^ 1st Department of Internal Medicine—Cardioangiology, St. Anne’s University Hospital Brno, Brno, Czech; ^6^ Biostatistics, International Clinical Research Center, St. Anne’s University Hospital Brno, Brno, Czech; ^7^ Division of Cardiology and Structural Heart Diseases, Medical University of Silesia, Katowice, Poland; ^8^ Institute of Scientific Instruments of the Czech Academy of Sciences, Brno, Czech; ^9^ Department of Biology, Faculty of Medicine Masaryk University Brno, Brno, Czech; ^10^ Department of Pathology, University Hospital Brno, Brno, Czech

**Keywords:** evaluation of radiofrequency ablation lesions, comparison of methods, gross pathology inspection, high-resolution MRI evaluation, animal experiments, catheter ablation of arrhythmias, ex vivo MRI scanning

## Abstract

**Aims:** Gross pathology inspection (patho) is the gold standard for the morphological evaluation of focal myocardial pathology. Examination with 9.4 T magnetic resonance imaging (MRI) is a new method for very accurate display of myocardial pathology. The aim of this study was to demonstrate that lesions can be measured on high-resolution MRI images with the same accuracy as on pathological sections and compare these two methods for the evaluation of radiofrequency (RF) ablation lesion dimensions in swine heart tissue during animal experiment.

**Methods:** Ten pigs underwent radiofrequency ablations in the left ventricle during animal experiment. After animal euthanasia, hearts were explanted, flushed with ice-cold cardioplegic solution to relax the whole myocardium, fixed in 10% formaldehyde and scanned with a 9.4 T magnetic resonance system. Anatomical images were processed using ImageJ software. Subsequently, the hearts were sliced, slices were photographed and measured in ImageJ software. Different dimensions and volumes were compared.

**Results:** The results of both methods were statistically compared. Depth by MRI was 8.771 ± 2.595 mm and by patho 9.008 ± 2.823 mm; *p* = 0.198. Width was 10.802 ± 2.724 mm by MRI and 11.125 ± 2.801 mm by patho; *p* = 0.049. Estuary was 2.006 ± 0.867 mm by MRI and 2.001 ± 0.872 mm by patho; *p* = 0.953. The depth at the maximum diameter was 4.734 ± 1.532 mm on MRI and 4.783 ± 1.648 mm from the patho; *p* = 0.858. The volumes of the lesions calculated using a formula were 315.973 ± 257.673 mm^3^ for MRI and 355.726 ± 255.860 mm^3^ for patho; *p* = 0.104. Volume directly measured from MRI with the “point-by-point” method was 671.702 ± 362.299 mm^3^.

**Conclusion:** Measurements obtained from gross pathology inspection and MRI are fully comparable. The advantage of MRI is that it is a non-destructive method enabling repeated measurements in all possible anatomical projections.

## 1 Introduction

Gross pathological inspection (patho) and manual measurements are standard methods in autopsy evaluation of tissue (Song et al., 2017) including myocardial pathologies and radiofrequency ablation lesions (Nakagawa et al., 1995) (Dickfeld et al., 2007) (Krahn et al., 2018). Nevertheless, this method inadvertently destroys the sample, effectively preventing reevaluation and allowing only unidirectional slicing. Therefore, patho is limited to complex anatomical objects, the number of measured planes is limited, and the calculated volume is only approximate.

Magnetic resonance imaging (MRI) represents well established diagnostics non-invasive method that allow exact repeatable measurements of myocardial pathologies (Ishihara et al., 2007) (Braggion-Santos et al., 2013) (Suzuki et al., 2021). Standardly used 1.5 T and 3 T MRI allows visualization of myocardial pathologies including radiofrequency lesions “*in vivo*” (Dickfeld et al., 2007) (Badger et al., 2010) (Markman and Saman 2017) (Tofig et al., 2019) as well post-mortem. Moreover, high-resolution MRI systems are very promising and rapidly evolving imaging methods (Schneider et al., 2008) (Schneider et al., 2011) (Wech et al., 2016) (Ertürk et al., 2017) (Erturk et al., 2019) (Heo et al., 2019), which allow precise evaluation of the heart in unparalleled quality. Berte et al. described the successful use of 1.5T MRI for *in vivo* imaging of the hearts and 9.4 T MRI (Bruker) for explanted hearts in 2015, where they used high-resolution MRI for evaluation of the lesions after radiofrequency (RF) ablation in an ovine experiment (Berte et al., 2015).

RF ablation is a catheter-based non-pharmacological treatment of cardiac arrhythmias (Kalbfleisch and Langberg 1992). The principle of this method is destruction of the myocardium, which serves as an arrhythmogenic substrate with RF energy applied to the tip of the ablation catheter (Haines and Verow, 1990). RF energy creates limited thermal coagulation necrosis that heals as the non-conducting scar and treats arrhythmia (D. E. Haines and Watson 1989) (Haverkamp et al., 1989) (Nath et al., 1994) (O’Donnell and Nadurata. 2004) (D. Haines 2018). The radiofrequency lesion is histologically characterized by central thermal coagulation necrosis, where myocardial cells lose clear boundaries and transverse striping. Central necrosis is surrounded by a thin border zone consisting of a mix of healthy and damaged cells. Behind this border zone is a healthy myocardium (Delacretaz et al., 1999) (Gepstein et al., 1999) (Krahn et al., 2018). Catheter ablation of arrhythmias is a very fast-growing area of cardiology, and many experiments have been performed. To evaluate the effect of RF ablation, it is crucial to evaluate the extent of necrosis, synonymously called RF lesions. The standard method is gross pathology; however, MRI evaluation is becoming very promising as an alternative method.

Therefore, the aim of this study was to demonstrate that lesions can be measured on high-resolution MRI images with the same accuracy as on pathological sections and compare results from MRI and patho measurements of the lesions formed after experimental RF ablation in swine heart tissue.

## 2 Materials and methods

### 2.1 Permission

The animal experiment was approved by the ethics committee of the University of Veterinary and Pharmaceutical Sciences in Brno (approval number 12–2018) and by the Ministry of Education Youth and Sports (approval number MSMT-17402/2018–3). All procedures performed on pigs were in accordance with Directive 2010/63/EU of the European Parliament on the protection of animals used for scientific purposes.

### 2.2 Animal preparation

The animal experiment was carried out in cooperation with the Animal Center FNUSA-ICRC Brno. Ten female swine (weight, 50–55 kg; age, 6 months) were used. Each animal was weighed on a digital scale to determine the exact weight. Premedication before anesthesia was done by mixture of ketamine 2 mg/kg + xylazine 2 mg/kg + tiletamine 2 mg/kg + zolazepam 2 mg/kg by intramuscular (i.m.) application. After this, pigs were intubated and an intravenous cannula was inserted into the ear vein. Animals were kept under mechanical ventilation with 1.5% isoflurane. Amiodarone (5 mg/kg, i. m.) was applied by very slow application to suppress ventricular fibrillation. Heparin (7.000 I.U., intravenous application) was administered before sheats were inserted into the femoral veins and then was repeated in a dose of 3500 I.U every hour. The animal arterial blood pressure was measured invasively in femoral artery. An oximeter was placed on the tongue of the animal to monitor oxygen saturation. A temperature control probe was inserted into the animal esophagus to minimize the risk of thermal damage to the esophagus. At the end of the experiment, animals were sacrificed by intravenous application (in dosage 4–6 ml/50 kg) of special mixture intended for euthanasia in animals named—T61 (Intervet International B.V., Boxmeer, Netherland).

### 2.3 RF ablations and examination of hearts

All pigs underwent a standard RF protocol under general anesthesia—left ventricles were ablated on the septum, on the lateral wall, on the inferior wall, on the anterior wall and in the left ventricular outflow tract (LVOT) position with a different power setting for 1 min or up until “steam pop” appeared. After completion of the ablation protocol, the animal was sacrificed and moved to the pathology room. Hearts were removed from the thoracic cavity and flushed with ice-cold cardioplegic solution (St. Thomas solution) to relax the whole myocardium. The heart was then fixed in 10% formaldehyde. Hearts were scanned with a 9.4 T magnetic resonance system (Bruker BioSpec 182 94/30USR), equipped with a volume coil 1H 198/154 mm. *Ex vivo* MRI imaging was performed in cooperation with the Institute of Scientific Instruments of the Czech Academy of Sciences. Anatomical images were processed using ImageJ software, which is intended for scientific use and is freely accessible on the website at www.imagej.net (Schindelin et al., 2015) (Rueden et al., 2017). All measurements done in ImageJ software were done manually without using any automatic processing tools. After the hearts were scanned, they were cut into 3–5-mm-thick slices, which were placed side by side, photographed by the camera (Nikon DX AF-S NIKKOR), and analyzed using the same ImageJ software. We opted for this method of analysis because manual measurement of the pathology samples is associated with a risk of inhaling toxic formaldehyde fumes. Each lesion was measured three times and evaluated by two independent observations.

As the last step, the heart was removed out from formaldehyde, rinsed under running water and several lesions with characteristic parameters (without “steam pop” effect) were cut out from the heart, with an approximate volume of 1 × 1 × 0.5 cm. The samples were rinsed again under running water, dehydrated *via* ascending ethanol series and subsequently immersed in xylene. Then the samples were immersed in liquid paraffin, and paraffin blocks were prepared. Tissue blocks were sliced into sections, fixed on the histological glass, and stained with hematoxylin and eosin and Masson’s trichrome (to visualize the viability of the tissue). Masson’s trichrome stains central necrosis purple, healthy myocardium is stained red, and the connective tissue is stained blue. Hematoxylin and eosin staining was used for basic resolution. The cytoplasm is stained pink and nuclei are stained purple (Krahn et al., 2018). Slides were examined and photographed under a digital microscope (Leica DM 5000B, Leica Microsystems, Germany) at 25x, 50x and 200x magnification.

### 2.4 Measurement of lesions

**FIGURE 1:**
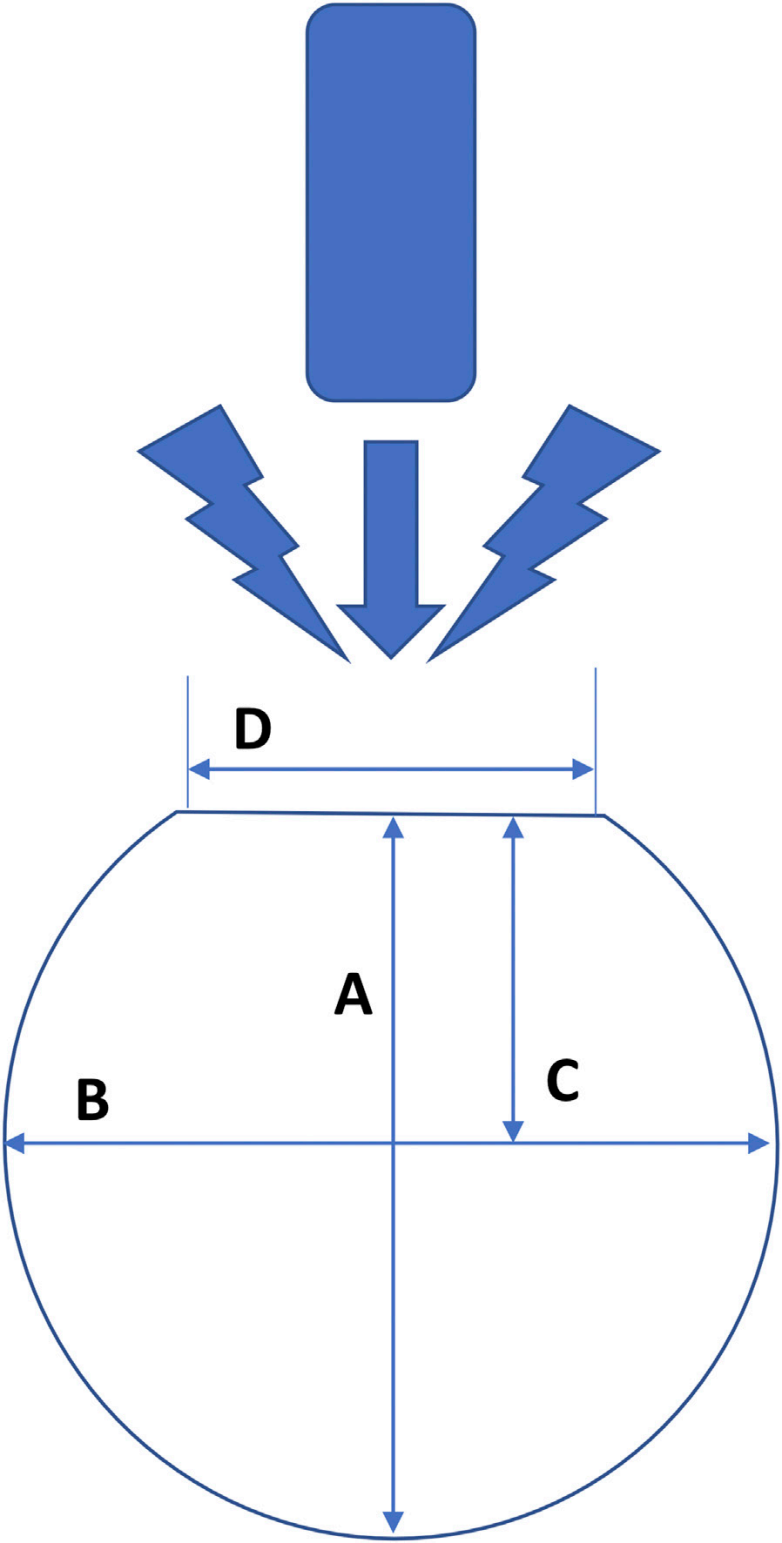
Schema of measuring the dimensions of lesions formed after radiofrequency ablation in the left ventricle of the swine hearts. **(A)** = maximum depth, **(B)** = maximum width, **(C)** = depth at the maximum diameter and **(D)** = lesion surface diameter (“estuary”). The measurement of each lesion takes place where the lesion reaches its maximum parameters.

*Depth* (A) of lesions was measured as the maximal distance between the point of contact of the catheter with the endocardium and the outermost apex of the lesion. *Width* (B) was measured in the place of the biggest length of the lesion. *Estuary* (D)—lesion surface diameter–was measured as a hollow in the endocardium created by catheter pressure. The *depth of the maximum diameter* (C) was measured as the distance between the *estuary* and the horizontal straight line passing through the widest point of the lesion (see Figure 1). The *volume* of the lesion was calculated from the measured parameters using the following formula (Guerra, 2013):

$$LV=\left(\left(0.75\pi \right)∙{\left(\frac{B}{2}\right)}^{2}∙\left(A-C\right)\right)-\left(0.25\pi ∙{\left(\frac{D}{2}\right)}^{2}∙\left(A-2C\right)\right)$$ (1)

### 2.5 Measurement of lesions on MRI scans

Hearts were scanned using the 9.4 T MRI system. Every sample was removed from fixation solution, rinsed with physiological solution (0.9% NaCl, Braun) to remove all formaldehyde and submerged in a plastic jar filled with clean physiological solution and underwent MRI scanning. During immersion of the samples into a physiological solution, a syringe was used to inject a solution into the atria and ventricles, to get rid of air bubbles and blood clots, which possibly could be the cause of MRI artifacts and reduce the quality of MRI slices. All hearts were scanned once. The regions of interest (ROIs) were left heart ventricles with ablation lesions. All MRI scans were performed on a 9.4 T Bruker BioSpec 94/30USR scanner with a Bruker volume coil 1H 198/154 mm. Fast low-angle shot (FLASH) scout images were used to localize the left ventricle with ablation lesions. Proton-density (PD) weighted anatomical images were taken using FLASH sequence with TR = 1268.9 ms, TE = 4.43 ms, FA = 45.7°, FOV 100 mm x 100 mm, 8 averages, and a 256 × 256 image matrix. Eighty axial slices with a thickness of 1.0 mm were acquired; the slices covered the whole heart. The raw MRI data format was exported to the DICOM format for the further analysis. These anatomical images provided sufficient background for optimal visualization of lesions and volumetric data analysis.

**FIGURE 2:**
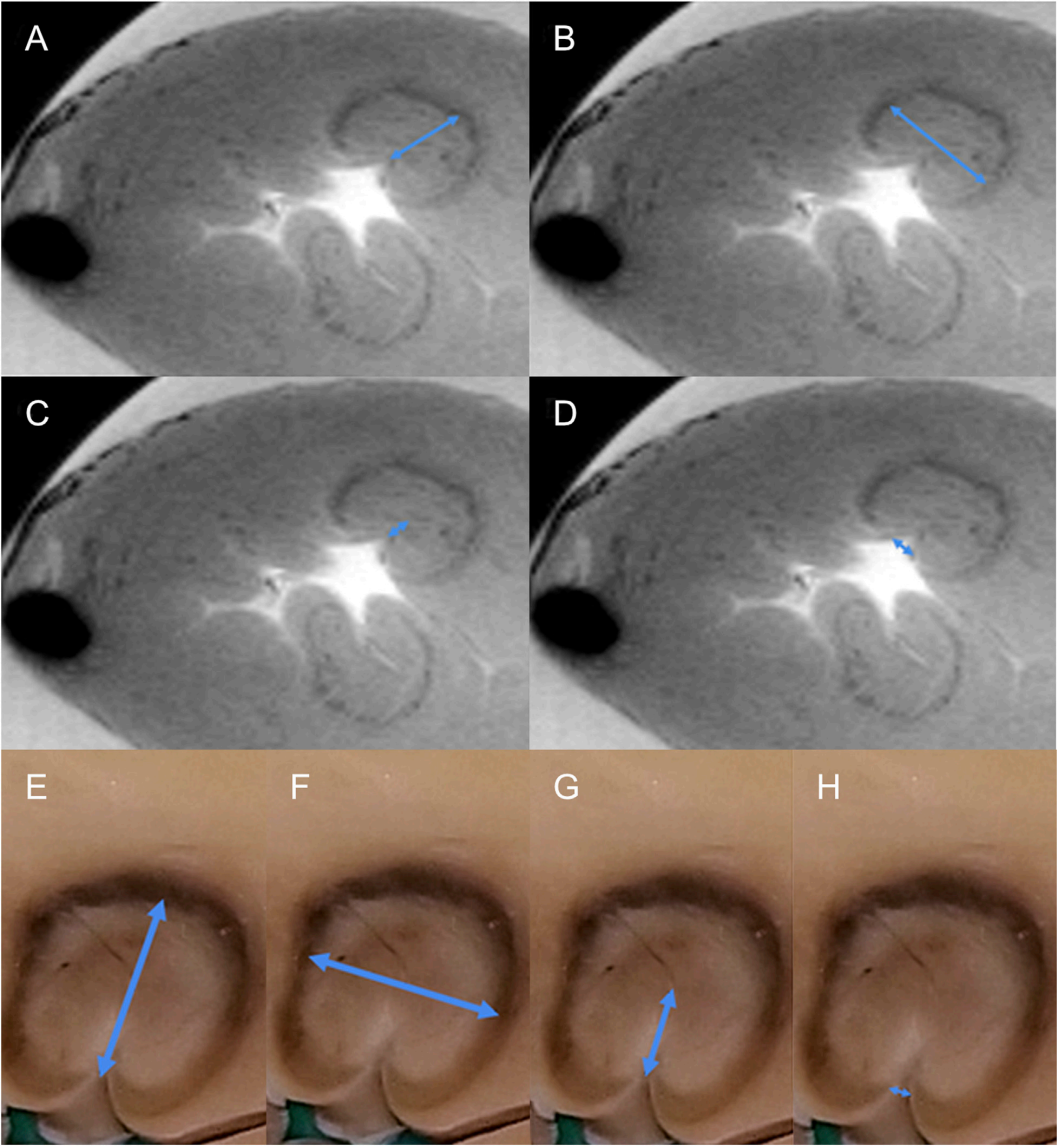
Example of measurement of the different dimensions of the radiofrequency lesion. **(A–D)**, measurement from MRI data, transverse section of the left ventricle. Two lesions are visible, the upper one is measured (blue arrows, A = depth, B = width, C = depth at the maximum diameter, and D = lesion surface diameter (“estuary). **(E–H)**, measurement from pathological samples, detail of the lesion, the same measurement as for MRI [blue arrows, E = depth, F = width, G = depth at the maximum diameter, and H = lesion surface diameter (“estuary”)].

Lesions were measured using ImageJ software. After image import, the ImageJ window was calibrated using the known pixel-to-mm ratio (1.024 pixel/mm). The measurement of each lesion took place in a slice, where the lesion reached its maximum parameters of d*epth* (A), *width* (B), *estuary* (D), and *depth at the maximum diameter* (C) (see Figure 1). Each lesion was bounded by a border zone that gave a low signal and was therefore displayed in black (Organ 1976) (Krahn et al., 2018). The border zone was a transition region between the necrotic, ablated and healthy, non-ablated myocardium (Ursell et al., 1985). We measured only the central completely necrotic part of the lesion. The border zone was not included in the measurement of lesion size (see Figure 2).

**FIGURE 3:**
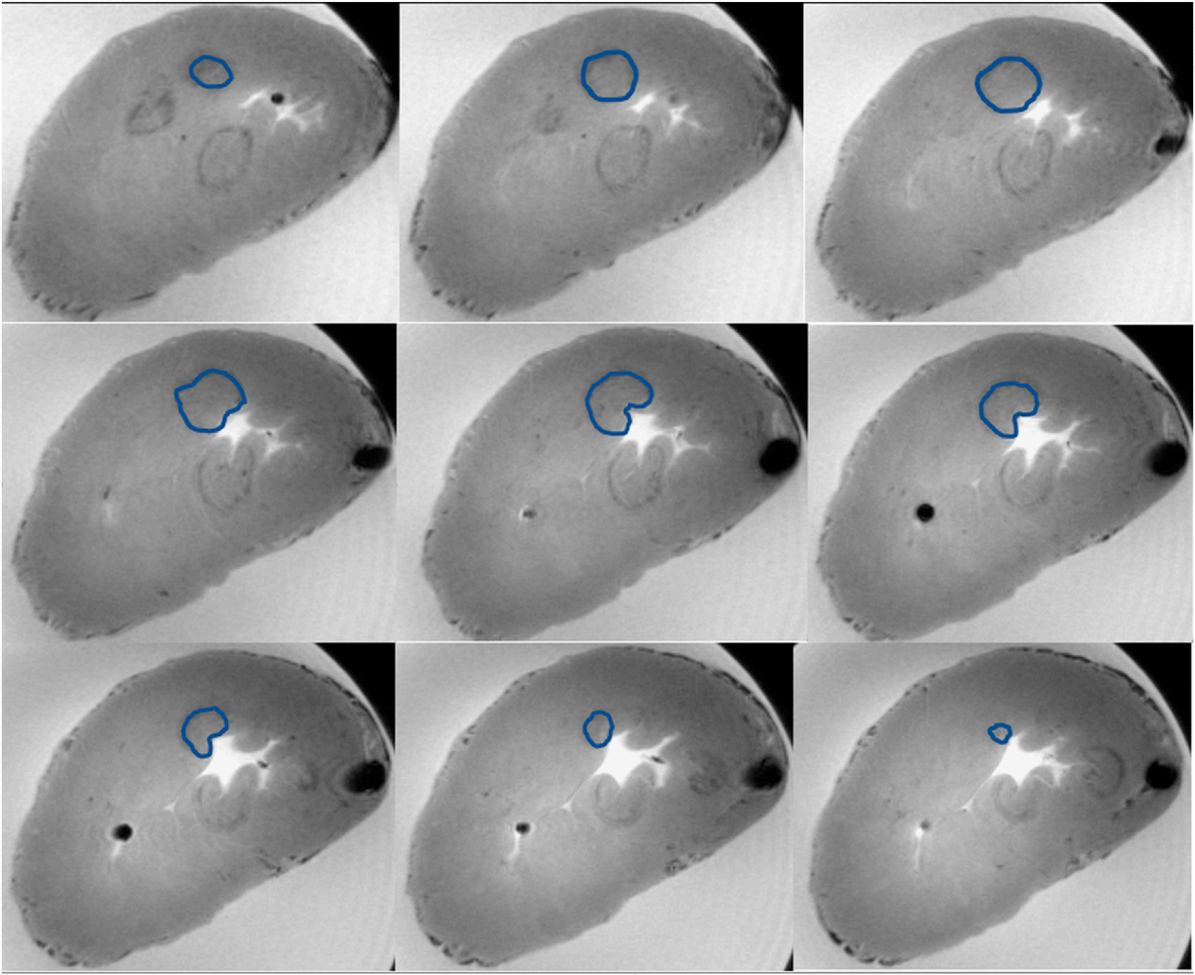
Example of volume measurement with the “point-by-point” method from MRI data. On the picture you can see nine subsequent transverse sections of the left ventricle capturing the whole radiofrequency lesion. Three lesions are visible, the upper one is measured. The measured lesion is outlined in all scans with a blue line. The resulting volume is the sum of the individual volumes from all involved MRI scans.

The *volume* of the lesions is calculated according to the abovementioned formula. In addition, we directly measured the lesion volume from MRI slices using the “point by point” method (see Figure 3) which is a variation of the Cavalieri principle. This method consists of gradually outlining the circumference of the lesion in all slices involving the measured lesion. The resulting volume is obtained by the total sum of all individually measured volumes from all slices where the lesion is displayed.

### 2.6 Measurement of lesions on pathology slices

**FIGURE 4:**
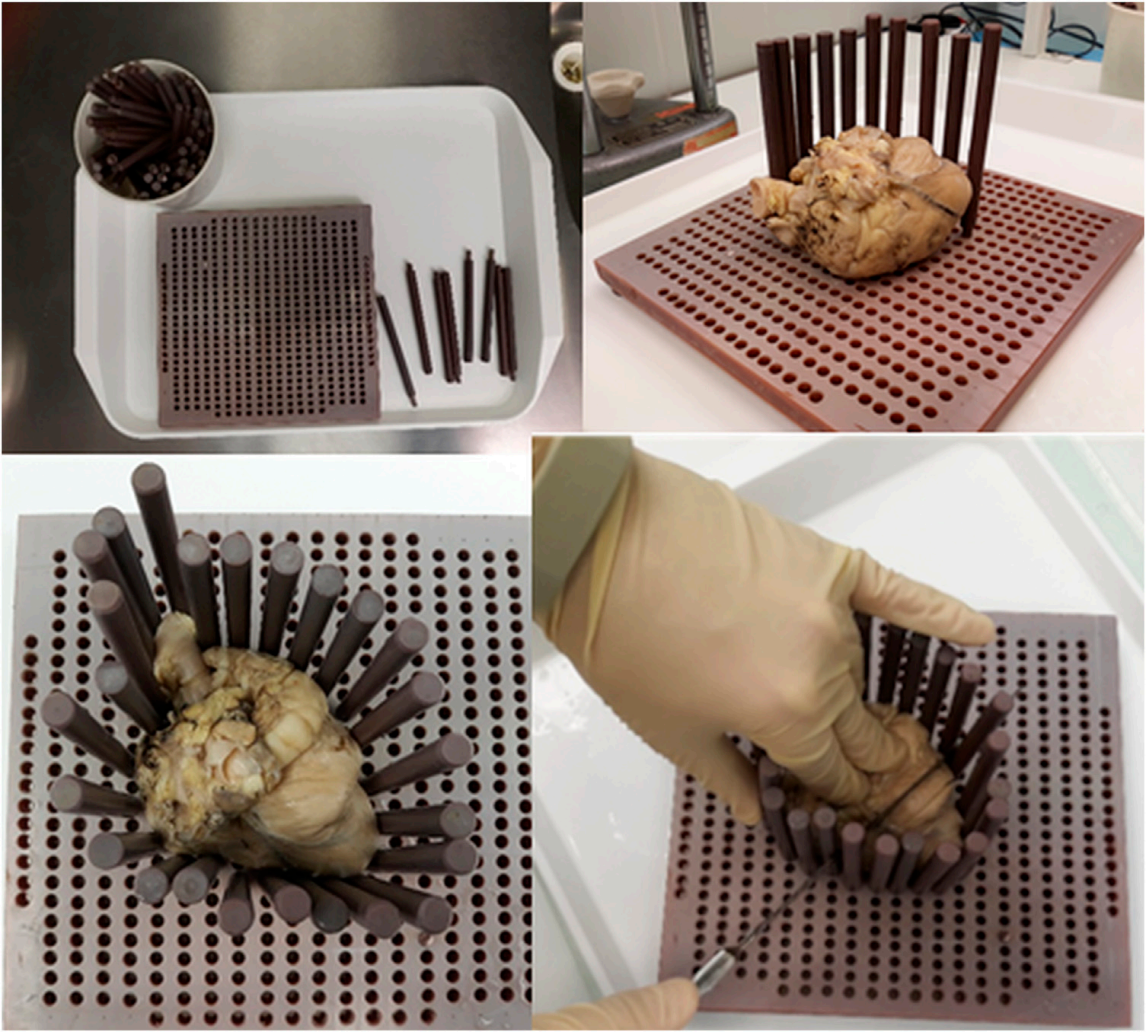
Use of the macrotome for slicing of the ventricles. In the basic plate are holes arranged in parallel rows. The swine heart is placed on the macrotome plate and fixed by pins all the way round. The heart is gradually sliced using a long sharp knife.

As a first step, the heart was manually sliced on the macrotome (see Figure 4), a device originally developed by Templeton in 1961 (Templeton 1961) for slicing brains during histopathological examinations. The macrotome consists of the following: *a base plate* made of plexiglass with dimensions 220 × 240 × 8 mm. In the plate, there are holes with a diameter of 5 mm, which are arranged in parallel rows. The distance between the holes is 1 cm in rows along the longer side of the plate and 0.7 cm in rows along the shorter side of the plate. The next parts of the macrotome are *fixing pins*, which are 11.8 cm long and have a diameter of 0.8 cm. One end of the *pins* is tapered to a diameter of 0.5 cm and is used to insert the pin into the plate. The swine heart is placed on the plate of the macrotome and is fixed by pins all the way round. Using a long sharp knife, the heart is gradually sliced.

**FIGURE 5:**
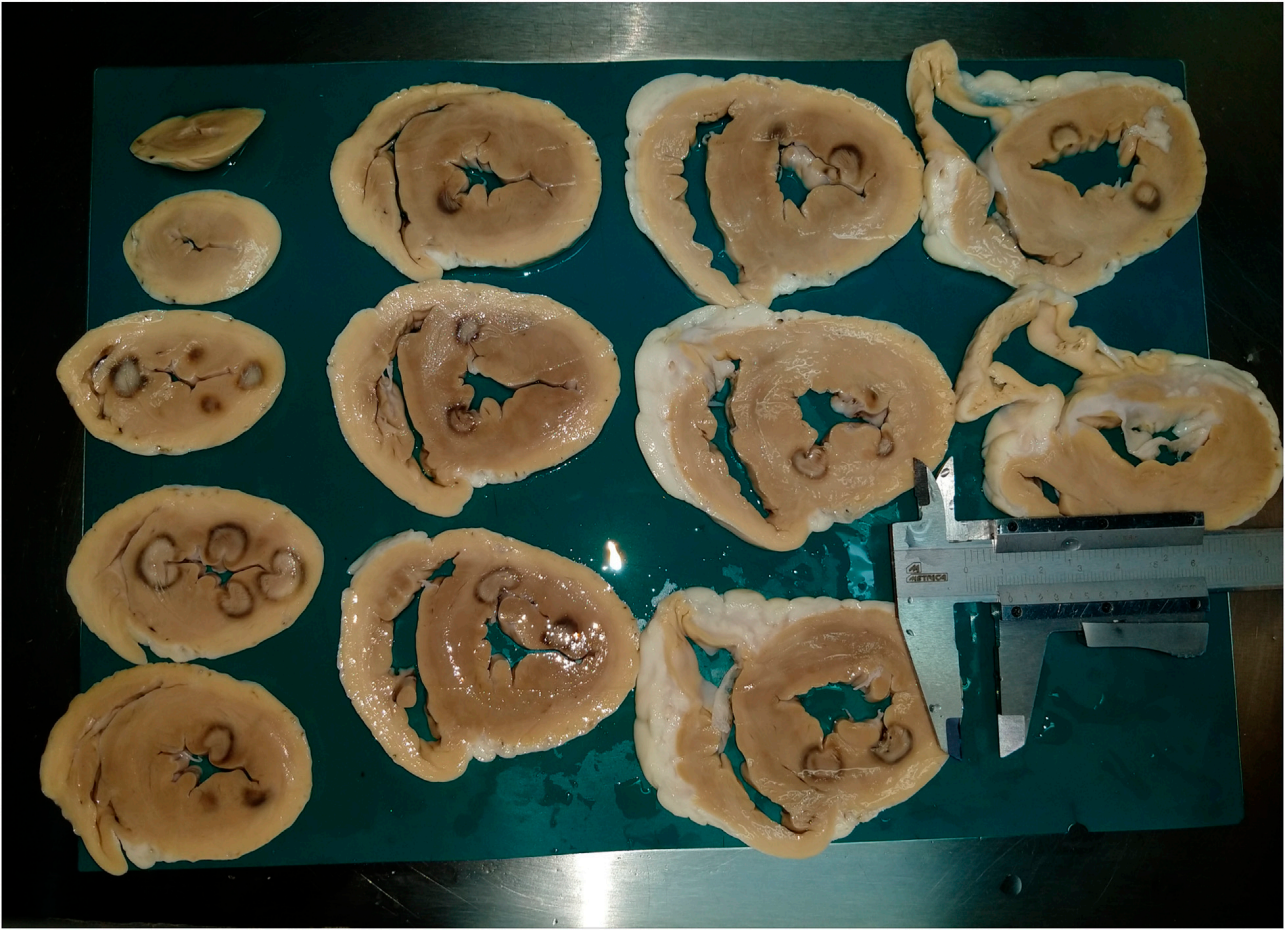
Example of a pathological picture used for measurement of radiofrequency lesions. On the picture you can see 13 subsequent transverse sections of the left ventricle. Radiofrequency lesions are visible. There is a caliper showing the exactly 1 cm, which was used to calibrate the software ImageJ.

Every heart was removed from the fixation solution, placed in a flow box, and manually sliced using the above-mentioned macrotome to 3–5-mm-thick slices. Slices were laid side by side, and pictures were taken using a camera (see Figure 5). All hearts were photographed in this way. The whole preparation of the heart for measuring pathological sections took approximately 20 min (fix the heart on the macrotome and slice it, take a photo, and upload it to ImageJ). Photos were imported to the ImageJ program. The calibration of the software was set manually according to the settings of the caliper, which is located on each photo and shows exactly the size of 1 cm. The *depth, width, estuary, and depth at the maximum diameter* were measured in the same way, as in MRI scans (from photo where is the lesion in maximal dimensions, black surrounding zone not included) (see Figure 2). *The volume* of the lesions was calculated using the abovementioned formula.

### 2.7 Statistical analysis

All variables are described as mean ± SD, if symmetrically distributed, otherwise as median (IQR). In order to compare measurements for all within-subjects factors—method (MRI, patho), observer (two independent persons), and measurement (all lesions were measured three times by each method and observer), least squares means from multilevel (mixed-effect) models were compared separately for depth, width, estuary, depth at the maximum diameter, volume calculated by formula, and for volume directly measured from MRI with the “point-by-point” method. No interactions among factors were considered. Additionally, in the case of variable width, the measurements were split into two separate models by a factor *observer* in order to analyze the data independently on the observer. In the analysis of the time of measurement, due to skewness, the data were first log-transformed and then a least squares means from multilevel (mixed-effect) model was used to compare the methods (MRI, patho) as a single within-subjects factor. Comparison of recognized lesions was performed using the chi-square test. All tests were performed at a significance level of α = 0.05. Statistical analysis was conducted using R version 3.6, RStudio version 1.2.1335, and package lme4 version 1.1–21.

## 3 Results

**FIGURE 6:**
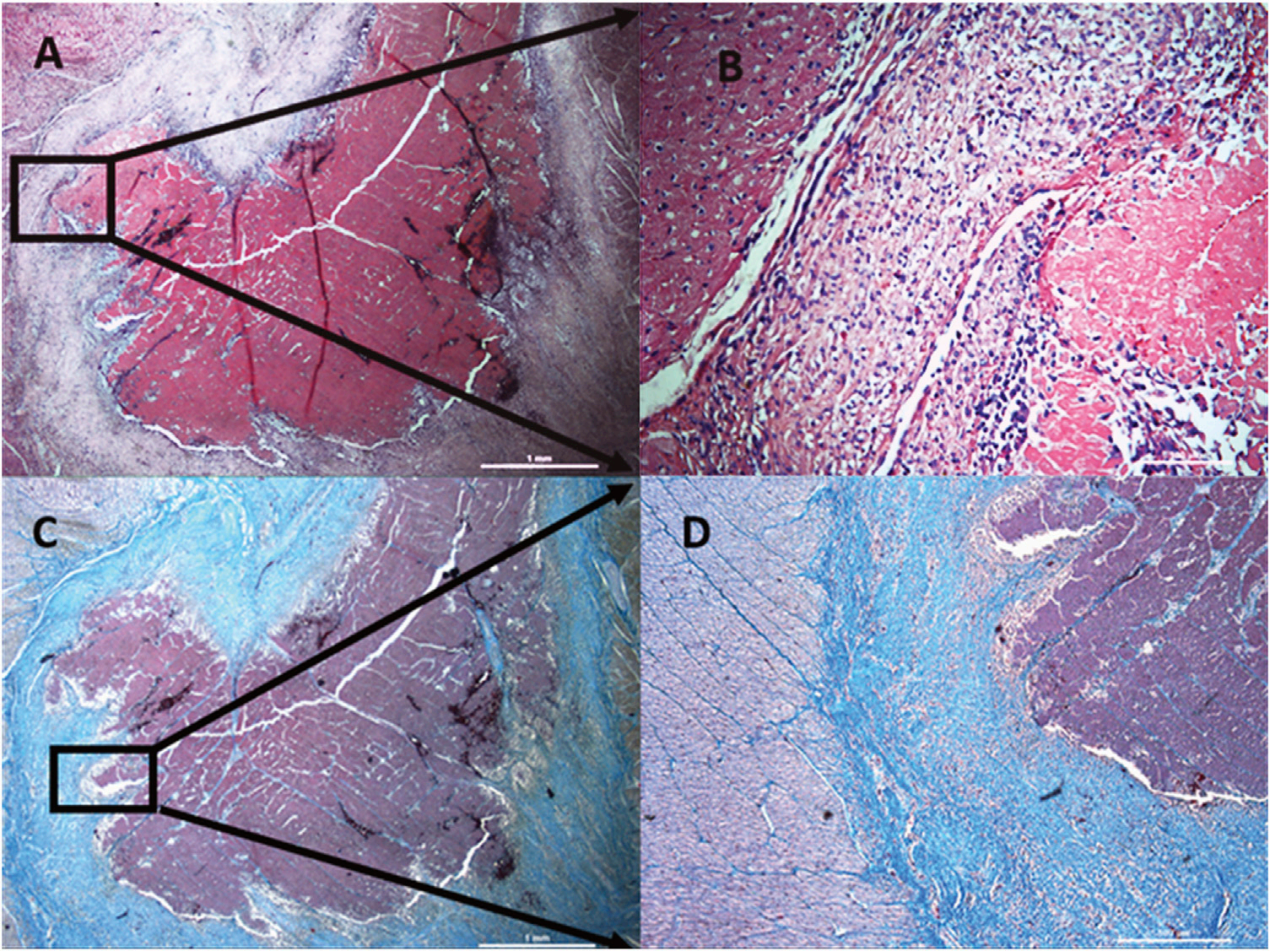
Histological characteristics of lesions created after radiofrequency ablation of porcine hearts. Lesions created by radiofrequency ablation are formed by coagulation necrosis, where cells have no borders and they are completely destroyed. Nuclei are not visible. Every lesion is surrounded by a differently wide rim consisting of cells at different stages of necrosis (damaged cell membrane and nuclei) sharply transitioning into healthy cardiomyocytes (clearly demarcated individual cells, pink cytoplasm, distinct purple nuclei). **(A)** hematoxylin-eosin staining, magnification 25x. **(B)** detail from **A**, magnification 200x, **(C)** trichrome staining, magnification 25x, **(D)** detail from **C,** magnification 50x.

Histological characteristics of lesions created after RF ablation of porcine hearts are signs of coagulative necrosis characterized by karyolysis with significant cytoplasmic hypereosinophilia. Each of the lesions was loosely demarcated by a narrow border zone consisting of cardiomyocytes in different stages of cell damage. Unaffected tissue displays no tissue damage and retains the conventional morphology of cardiac tissue (see Figure 6).

A total of 99 lesions were created; the MRI method successfully evaluated 92 (92.93% from all lesions), and with the patho method successfully evaluated 98 (98.99% from all lesions). No statistically significant difference was found (*p* = 0.755) between the two methods. *The depth* from MRI was 8.771 ± 2.595 mm, and from patho was 9.008 ± 2.823 mm; *p* = 0.198. *The width* of the lesion was 10.802 ± 2.724 mm on MRI, and 11.125 ± 2.801 mm with patho; *p* = 0.049. The limit *p*-value for the variable width is created by the interpersonal variability. Splitting models into two independent structures, we observed no statistically significant effect of method. The lesion values were almost identical, showing 2.006 ± 0.867 mm by MRI and 2.001 ± 0.872 mm by patho; *p* = 0.953. The *depth at the maximum diameter* was 4.734 ± 1.532 mm in MRI slices and 4.783 ± 1.648 mm from patho slices, *p* = 0.858. The *volumes* of the lesions calculated using a formula from both methods were 315.973 ± 257.673 mm^3^ for MRI and 355.726 ± 255.860 mm^3^ for patho; *p* = 0.104. *Volume* acquired from MRI by the “point-by-point” method was 671.702 ± 362.299 mm^3^, which is two times larger than the volumes from the patho or MRI calculated using a formula (Table 1).

**TABLE 1:** Comparison of evaluation of radiofrequency lesions with gross pathology inspection (patho) and MRI evaluation.

Variable	MRI	Patho	*p* value
Number of recognised lesions (N = 99)^a^	92 (92.93%)	98 (98.99%)	*p* = 0.755
Depth (mm)^b^	8.771 ± 2.595	9.008 ± 2.823	*p* = 0.198
Estuary (mm)^b^	2.006 ± 0.867	2.001 ± 0.872	*p* = 0.953
Depth at the maximum diameter (mm)^b^	4.734 ± 1.532	4.783 ± 1.648	*p* = 0.858
Volume by formula (mm3)^b^	315.973 ± 257.673	355.726 ± 255.860	*p* = 0.104
Volume by “point-by-point” method from MRI (mm3)^b^	671.702 ± 362.299	NA	NA
Width (mm)^b^	10.802 ± 2.724	11.125 ± 2.801	*p* = 0.049
Width splitted by observer			
Observer 1^c^	10.202 ± 2.826	10.620 ± 2.864	*p* = 0.086
Observer 2^c^	11.400 ± 2.484	11.629 ± 2.646	*p* = 0.299

a - comparison of number of recognizable lesions.

b - mean ± standard deviation for patho and MRI measurements. Comparison of methods was performed by comparing least squares means from mixed-effect models.

c - width data (with borderline statistical significance) were split by observer and two separate mixed-effect models were used to compare the MRI and patho methods.

The complete time for preparing and MRI scanning was 115 ± 3 min (cleaning the heart from formaldehyde, placing it into the physiological solution, setting the MRI device, scanning the MRI sample, and controlling the quality of MRI scans after scanning). Preparing the heart for patho measurement, cutting of the slices on the macrotome for patho measurements and taking photos, took 21 ± 1 min.

The difference between the duration of measurements from MRI slices and from patho as follows: for MRI measurement, the median value was 56 s (53–59.2), whereas the median value for patho was 53 s (47–59), *p* = 0.001. Statistically significant differences were not found for *intrapersonal variability* (Supplementary File S1), but for i*nterpersonal variability* between observer 1 and observer 2, respectively, as follows: *width* was 10.418 ± 2.851 mm and 11.518 ± 2.569 mm (*p* < 0.0001), *depth* was 8.501 ± 2.937 mm and 9.286 ± 2.416 mm (*p* < 0.0001), *estuary* was 2.055 ± 0.867 mm and 1.942 ± 0.870 mm (*p* = 0.042), and *depth at the maximum diameter* was 4.638 ± 1.621 mm and 4.909 ± 1.558 mm (*p* = 0.002), respectively.

## 4 Discussion

To the best of our knowledge, this is the first study focused on the comparison of two measuring methods (MRI x patho) of ablation lesions in the left ventricle. We compared two possible methods for measuring the dimensions of lesions formed in the myocardium of ventricles after RF ablation in an animal experiment. Our results showed, like the other studies (Lardo Albert et al., 2000; Dickfeld et al., 2007), that measurements obtained from pathology gross inspection (patho) and from MRI are fully comparable. For the variabiles *depth, estuary, depth at the maximum diameter*, and *volume by formula*, we found no statistically significant difference between these two methods. The limit *p*-value for the variable *width* was created by the interpersonal variability. Splitting models into two independent structures, we observed no statistically significant effect.

**FIGURE 7:**
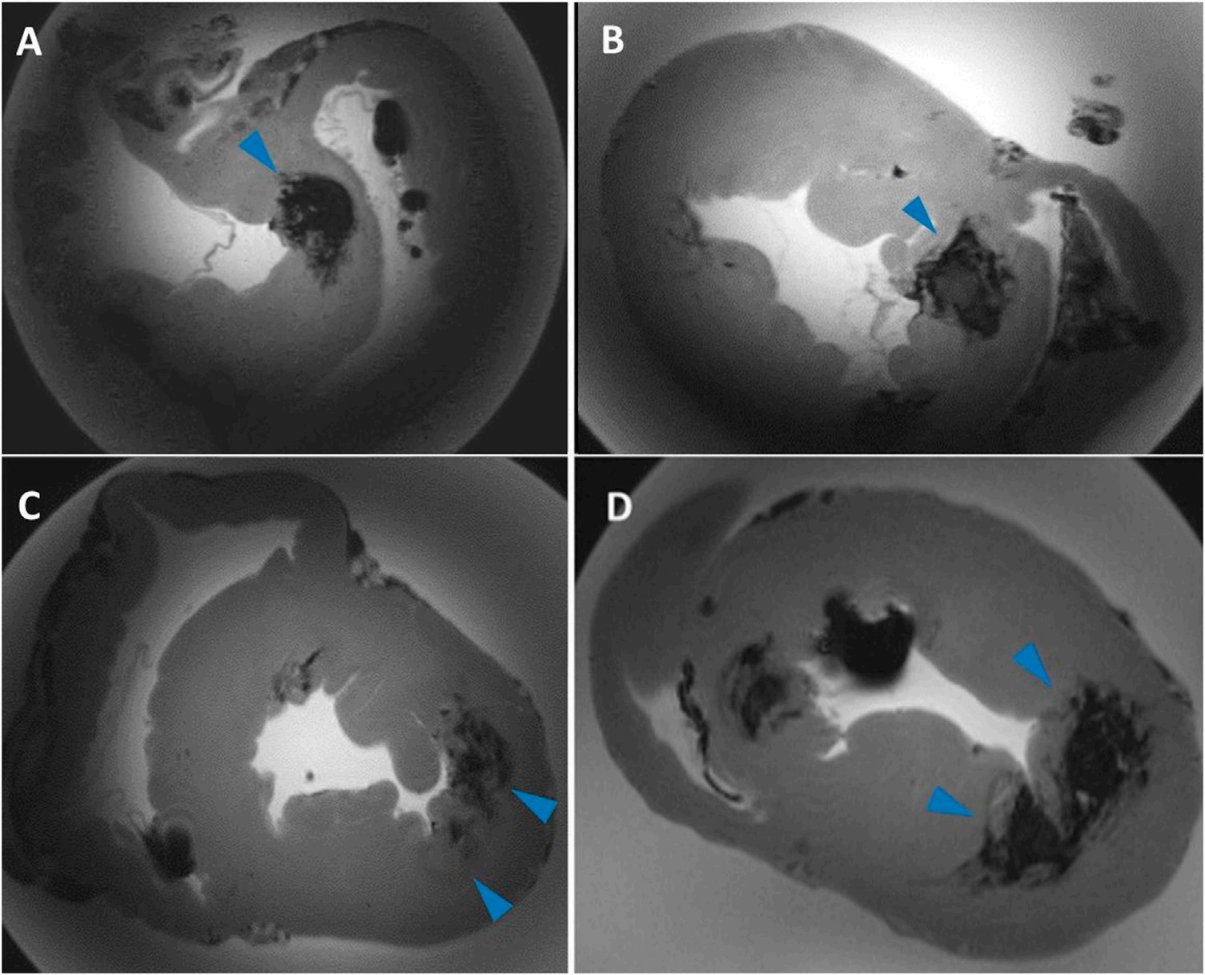
Examples of complex lesions. Transverse sections of ventricles of the swine heart on MRI scans. **(A,B)** shows the irregular shape of lesions with unclear borders. During high energy applications, a “steam pop” sometimes occurs, which causes rupture of the myocardial tissue. This rupture spreads into the myocardium and causes irregular and badly bounded lesions. **(C,D)** example of fused lesions—lesions formed so close each other that they partially merge into one.

We found a statistically significant difference in values for directly measured *volume* from MRI (“point-by-point” method) and *volume* calculated by formula from MRI slices or patho. The reason for this is the more precise characteristics of the “point-by-point” method, where the *volume* was measured as a summary of all partial volumes obtained by manual gradual outlining of the lesion edges from all involved MRI slices. Another method of determining the lesion *volume* is to use a formula; a prerequisite for successful use of the formula is a regular oval shape of the lesion, which cannot be fully achieved in RF ablation lesions. RF lesions with higher energy settings are not precisely delineated and have a completely irregular shape (Figure 7). Therefore, using a formula to calculate volume has not been successful due to the irregular shape of the lesions. From this point of view, it seems more precise to use the “point-by-point” method.

Similar to previous studies (Dinkel et al., 2013) (Bolte et al., 2007) (Zhao et al., 2013) (Thiesse et al., 2016) (Erasmus et al., 2003) (Muenzel et al., 2012) (Lazebnik et al., 2005) we found statistically significant differences in all measured parameters for *interpersonal differences* between both observers. This can be explained by the high-power values that were used and that we had several “steam pop” effects. If a “steam pop” effect occurred, there was a partial disruption of the myocardium, which spreads to the width and not to the depth of the tissue. For complex lesions such as RF lesions, is not easy to pinpoint the maximal parameters of the lesion after “steam pop” (Figure 7). Another possible explanation for the statistically significant difference in *interpersonal differences* is an individual assessment on which MRI slice the lesion reaches its maximum size. In addition, on several occasions, the individual lesions were placed close together such that they partially fused together (Figure 7). In this case, it depended on the individual evaluation of each observer as to where was the exact border of every lesion was.

No statistically significant difference was found in *intrapersonal variability.* This indicates the reproducibility of both measurement methods (see Supplementary File S1).

An important parameter for the evaluation of lesions is the time needed to perform a single measurement. We compared how long it takes to measure the basic parameters of the lesion (depth, width, estuary, and depth at the maximum diameter) from MRI slices and patho samples. The difference between the duration of measurements from MRI slices (without gradual outlining) and from patho was statistically significant (*p* = 0.001). The median value for MRI measurement was 56 s (53–59.2) and for patho measurement was 53 s (47–59). The difference between the duration of the measurement is significant from a statistical point of view; however, a difference of 3 s is from a practical point of view is negligible.

If outlining by the “point by point” method is included in the time measurement, the measurement of the lesion from MRI slices is significantly longer. In particular, the duration was 235.5 s (201–268.25) for overall MRI measurement and 177.5 s (144.8–208.5) for “point-by-point only.” This increase in time is fully compensated by the higher accuracy of the “point-by-point” method.

In our work, we recognized 92.93% of the lesions on MRI scans compared with 75% published by (Berte et al., 2015). In our study, were created 99 lesions in total; 92 lesions (92.93%) were identified by MRI and 98 (98.99%) lesions were identified by patho. One lesion could not be traced on patho or MRI, which represents 1.01%. Three lesions were not recognized in one heart as they were covered by a single MRI artifact and could not be identified in the first scan, representing 3.03% of all lesions. Three additional lesions were identified by patho, but not recognized on MRI. These lesions were small and did not have high intensity border with an overall “ghost-like” character.

In our study, hearts underwent MRI scanning submerged in physiological solution (0.9% NaCl, Braun), which is easy to handle and obtain. On the other hand, there is a background signal on MRI scans; thus, the image background of the scan is gray and produces suboptimal contrast. The use of Fomblin Y06/6 is recommended by other authors for optimal outcomes (Bulte et al., 2003) (Lipinski et al., 2006).

In Figure 7 there is a certain inhomogeneity of the image. The shading and the brightness difference in the center and the periphery of the image is an MRI artifact occurring during the scanning of larger dimension samples. Since the hearts were not exactly the same shape and size, the artifact occurred only by some of them. This artifact did not interfere with the lesions, thus did not obstruct the image processing, and did not affect the results. Therefore we did not use any filter. For the future, we are working on the filter creating and applying it on such sample scans.

A limitation of our study is that it is an *ex vivo* model on a non-beating pig heart. Also evaluation is limited in visualization of small, not very well-demarcated lesions. As well, preparing of the heart before MRI scanning and the scanning time itself is longer than that of gross pathology inspection. Furthermore, access to a 9.4 T MRI device is required. Not negligible is also the purchase price of MRI machine and the price of the organ scanning. As the limitation of our study must be also mentioned statistically significant difference in interpersonal differences in our case. Interpersonal differences will decrease with the gradually increasing experience of the observes.

## 5 Conclusion

Measurements obtained from pathology gross inspection and MRI scanning did not significantly differ in absolute values; however, MRI allows a significantly more precise assessment of lesion volume. The measurement time of the lesions on MRI was significantly longer from the statistical point of view, but negligible from a practical point of view. MRI evaluation is a nondestructive method, allowing for diverse scanning angles, resulting in clear lesion definition and more precise volume assessment.

## Data Availability

The original contributions presented in the study are included in the article/Supplementary Materials, further inquiries can be directed to the corresponding author.
