# Supplementary material for: Comparison of gross pathology inspection and 9.4 T magnetic resonance imaging in the evaluation of radiofrequency ablation lesions in the left ventricle of the swine heart
Source: Front Physiol. 2022 Oct 19;13:834328. doi: 10.3389/fphys.2022.834328 (PMC9626654; doi:10.3389/fphys.2022.834328)
Supplement: Supplementary file 1 [file DataSheet1.docx]

**Supplementary material**

Table 1: Mean ± standard deviation – interpersonal variability. Comparison of observers was performed by comparing least squares means from mixed-effect models.

| **Variable** | **Observer 1** | **Observer 2** | **p value** |
| --- | --- | --- | --- |
| **Depth** | 8.501 ± 2.937 | 9.286 ± 2.416 | p<0.0001 |
| **Width** | 10.418 ± 2.851 | 11.518 ± 2.569 | p<0.0001 |
| **Estuary** | 2.055 ± 0.867 | 1.942 ± 0.870 | p= 0.042 |
| **Depth at the maximum diameter** | 4.638 ± 1.621 | 4.909 ± 1.558 | p= 0.002 |
| **Volume by formula** | 304.864 ± 270.081 | 378.122 ± 235.305 | p <0.0001 |
| **Volume from MRI by "point-by-point" method** | 459.994 ± 357.502 | 566.805 ± 364.729 | p <0.0001 |

Table 2: Mean ± standard deviation – intrapersonal variability. Comparison of measurements was performed by comparing least squares means from mixed-effect models.

| **Variable** | **Measurement 1** | **Measurement 2** | **Measurement 3** | **p value** |
| --- | --- | --- | --- | --- |
| **Depth** | 8.916 ± 2.731 | 8.855 ± 2.681 | 8.909 ± 2.743 | p= 0.922 |
| **Width** | 11.126 ± 2.867 | 10.852 ± 2.767 | 10.928 ± 2.665 | p = 0.233 |
| **Estuary** | 2.033 ± 0.863 | 1.975 ± 0.879 | 2.008 ± 0.867 | p= 0.807 |
| **Depth at the maximum diameter** | 4.738 ± 1.629 | 4.775 ± 1.576 | 4.766 ± 1.599 | p= 0.993 |
| **Volume by formula** | 352.573 ± 292.198 | 329.876 ± 249.953 | 335.844 ± 235.617 | p = 0.241 |
| **Volume from MRI by "point-by-point" method** | 564.322 ± 381.913 | 479.353 ± 355.538 | 496.761 ± 356.601 | p= 0.242 |
